# *Salvianolic acid B* in fibrosis treatment: a comprehensive review

**DOI:** 10.3389/fphar.2024.1442181

**Published:** 2024-07-30

**Authors:** Qingzhi Liang, Xiaoqin Liu, Xi Peng, Ting Luo, Yi Su, Xin Xu, Hongyan Xie, Hong Gao, Zhengtao Chen, Chunguang Xie

**Affiliations:** ^1^ Hospital of Chengdu University of Traditional Chinese Medicine, Chengdu, Sichuan, China; ^2^ TCM Regulating Metabolic Diseases Key Laboratory of Sichuan Province, Chengdu, Sichuan, China; ^3^ Department of Endocrinology, Hospital of Chengdu University of Traditional Chinese Medicine, Chengdu, Sichuan, China; ^4^ Affiliated Hospital of Jiangxi University of Chinese Medicine, Nanchang, Jiangxi, China

**Keywords:** *Salvianolic acid B*, fibrosis, natural product, pharmacological mechanism, treatment

## Abstract

Fibrosis is a public health issue of great concern characterized by the excessive deposition of extracellular matrix, leading to the destruction of parenchymal tissue and organ dysfunction that places a heavy burden on the global healthcare system due to its high incidence, disability, and mortality. *Salvianolic acid B* (*SalB*) has positively affected various human diseases, including fibrosis. In this review, we concentrate on the anti-fibrotic effects of *SalB* from a molecular perspective while providing information on the safety, adverse effects, and drug interactions of *SalB.* Additionally, we discuss the innovative *SalB* formulations, which give some references for further investigation and therapeutic use of *SalB*’s anti-fibrotic qualities. Even with the encouraging preclinical data, additional research is required before relevant clinical trials can be conducted. Therefore, we conclude with recommendations for future studies. It is hoped that this review will provide comprehensive new perspectives on future research and product development related to *SalB* treatment of fibrosis and promote the efficient development of this field.

## 1 Introduction

**FIGURE 1:**
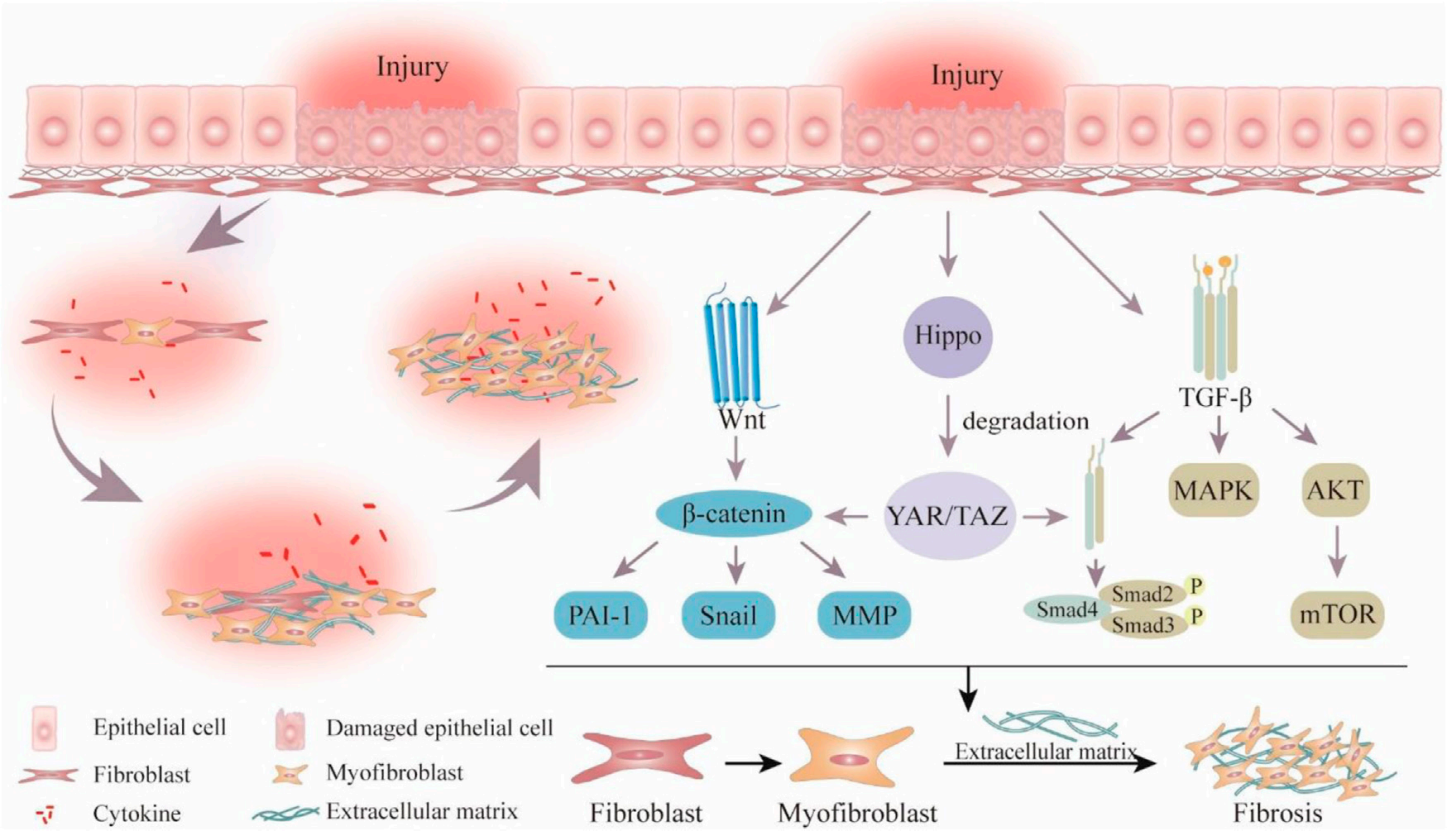
Schematic overview of pathological process and signaling pathways related to fibrosis. Wingless/Integrated (Wnt), plasminogen activator inhibitor-1 (PAI-1), matrix metalloproteinase (MMP), Yes-associated protein 1 (YAP), transcriptional coactivator with PDZ-binding motif (TAZ), transforming growth factor-β (TGF-β), mitogen-activated protein kinase (MAPK), the protein kinase (AKT), mechanistic target of rapamycin (mTOR).

Fibrosis is characterized by the destruction of fibrous connective tissue, activation and proliferation of fibroblasts, elevated collagen fiber secretion, and excessive deposition of extracellular matrix (ECM) (Zhang and Zhang, 2020). Activation of myofibroblasts, which produce ECM as effector cells in fibrotic illnesses, is a notable feature of these conditions (Wynn and Ramalingam, 2012; Walraven and Hinz, 2018). According to reports, the stimulation and maintenance of the myofibroblast phenotype are dependent on the transforming growth factor-β (TGF-β)/Smad, wingless/Integrated (Wnt), and yes-associated protein 1 (YAP)/transcriptional coactivator with PDZ-binding motif (TAZ) signaling pathways (Figure 1) (Piersma et al., 2015; Ricard-Blum and Miele, 2020). Notably, excessive deposition of ECM within organs leads to the destruction or replacement of parenchymal tissue, irreversible scarring, and organ dysfunction or failure, such as heart failure, chronic renal failure, and chronic pancreatitis (King et al., 2011; Weiskirchen et al., 2019). Currently, Nidanib and Pirfenidone are the only approved drugs for treating idiopathic pulmonary fibrosis (IPF). However, they carry the risk of gastrointestinal and dermatological adverse reactions and cardiovascular events (Henderson et al., 2020). Furthermore, there is currently no specific treatment strategy for other tissue fibrosis (Rosenbloom et al., 2017). Therefore, it is extremely urgent to investigate efficient and safe anti-fibrotic therapeutic approaches.

**FIGURE 2:**
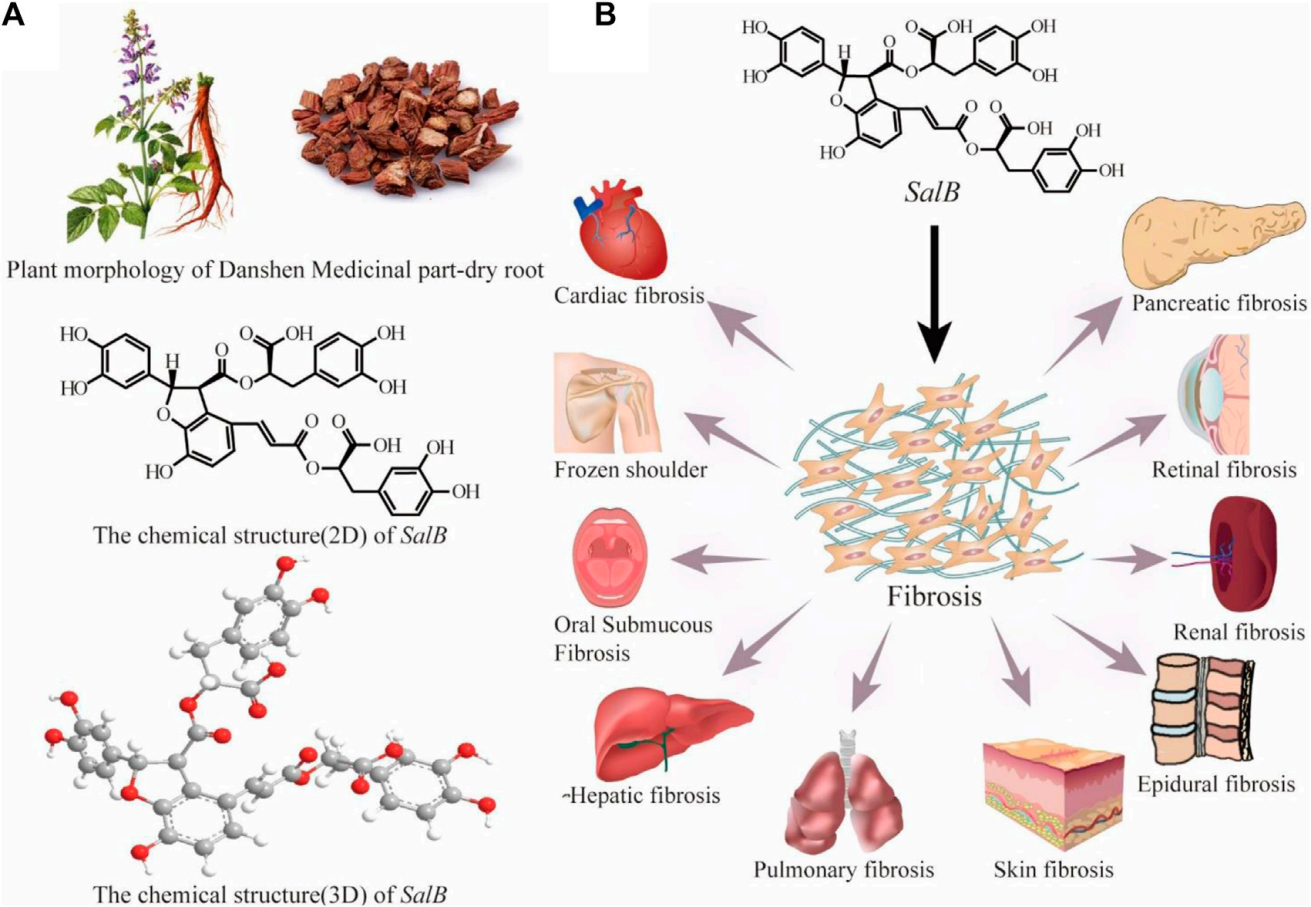
Plant morphology and medical part-dry root of Danshen and the chemical structure (2D and 3D) of *SalB* **(A)**; *SalB* is effective for numerous fibrosis diseases **(B)**.

Natural products are uniquely suited to treat fibrotic disorders because of their high level of safety and precise efficacy (Wang Y. et al., 2024; Deng et al., 2024; Liu et al., 2024; Xing et al., 2024). It not only adjusts immune function but also lessens side effects from other medications, slows the growth of fibrosis, and greatly improves the quality of life for patients. Danshen, the dried root of rhizome of *Salvia miltiorrhiza* Burge, a perennial herb in the genus Salvia, family Labiatae, was originally recorded in *Shennong Bencao Jing*. The main bioactive monomeric component of the hydrophilic compounds of Danshen is *salvianolic acid B*(*SalB*) (Figure 2A), which is produced through the condensation of one molecule of caffeic acid and three molecules of *salvianic acid A* (Liu et al., 2007). Modern pharmacological research has found that *SalB* has anti-oxidant, anti-apoptotic, anti-inflammatory, and anti-fibrotic effects (Guo and Wang, 2022; Liu et al., 2023a; Yan et al., 2023; Zhou et al., 2023). Moreover, it exerts protective effects on various organs and tissues, such as the heart, liver, kidney, lung, skin, etc. (Figure 2B) (Cao et al., 2012; Jia et al., 2019). Consequently, it has drawn a lot of attention. Numerous studies have clarified *SalB*’s potential therapeutic effectiveness in a wide range of pathological conditions, including but not limited to myocardial infarction, membranous nephropathy, ischemic brain injury, retinal defects, intervertebral disc degeneration, diabetes mellitus, sepsis, and various other ailments (Su et al., 2020; Chen et al., 2022a; Zhang F. X. et al., 2022; Hu et al., 2022; Yan M. et al., 2023; Wang et al., 2023).

Despite extensive confirmation of *SalB*’s anti-fibrotic pharmacological action, no comprehensive literature has been produced to summarize this effect and related mechanisms. Therefore, Studies related to the anti-fibrotic effects of *SalB* published in the last 23 years were identified through major scientific databases (PubMed, Web of Science, Embase, Google Scholar). Additional articles were identified through citation tracking or by visiting journal websites. Keywords used during the search included *salvianolic acid B*, fibrosis, fibrotic disease, anti-fibrotic effects, pharmacological effects, pharmacological mechanism, safety, drug interactions, combination therapy, and new dosage.

## 2 Anti-fibrotic effect of *SalB*

### 2.1 Hepatic fibrosis

Hepatic fibrosis (HF) is a common pathological feature seen in many etiologies of chronic liver disease and is an intermediate stage in the disease’s progression (Tacke and Trautwein, 2015). It is caused by excessive deposition of type I and type III collagen-rich ECM in the liver, resulting in the formation of fibrous scars. (Tacke and Trautwein, 2015; Zhao et al., 2016). Inhibiting hepatic fibrogenesis is crucial for successful prevention and therapy methods against chronic liver illnesses, as numerous studies have shown the possibility of reversing liver injury at different stages of fibrosis (Lee et al., 2015). Pro-fibrotic factors secreted by hepatic stellate cells (HSC) aid in the creation of collagen by bone marrow-derived fibroblasts and myofibroblasts, which in turn contributes to the development and progression of HF (Trautwein et al., 2015; Zhang C. Y. et al., 2016). It is imperative to inhibit the proliferation, activation, and migration of HSC for effective HF prevention (Higashi et al., 2017).

#### 2.1.1 TGF-β

TGF-β is the most effective cytokine for sustaining HF due to its pro-fibrotic effects, and it is also an essential inducer of ECM formation (Tsuchida and Friedman, 2017). *SalB* has demonstrated its effectiveness in reducing diethyl nitrosamine-induced HF via altering the TGF-β/Smad and mitogen-activated protein kinase (MAPK) signaling pathways (Wu et al., 2019). Additionally, *SalB* downregulates the expression of the fibrosis gene plasminogen activator inhibitor-1 (PAI-1) and enhances protein levels of α-smooth muscle actin (α-SMA) and collagen type I (CoI), both indicative markers associated with HF. Furthermore, enhancer factor 2 (EF2) acts as a downstream effector within the TGF-β1 pathway and plays an essential role in HSC activation and the progression of fibrosis. Zhang et al. found that *SalB* exerts an anti-fibrotic effect by antagonizing TGF-β1-induced activation of myocyte enhancer factor 2 (MEF2) at protein and RNA levels (Zhang et al., 2019). Additionally, TGF-β1 promotes fibrosis by enhancing autophagic flux through increasing cellular autophagosomes, thus reducing the level of autophagy in fibrotic tissues, which may be a potential target for anti-fibrotic therapy (Weiskirchen and Tacke, 2019; Ye et al., 2020). Potent anti-fibrotic agent *SalB* suppressed autophagosome formation and autophagic flux in HSC by down-regulating the extracellular regulated protein kinases (ERK), c-Jun N-terminal kinase (JNK), and p38-MAPK pathways, leading to decreased expression of light chain 3β II, autophagy-related gene 5 (Atg5), α-SMA, and CoI (Jiang et al., 2022). To sum up, *SalB* might be a useful and potent TGF-β antagonist to postpone the development of HF.

#### 2.1.2 PDGF

Platelet-derived growth factor (PDGF) is a significant pathway to promote HF (Roehlen et al., 2020). Fibroblasts produce PDGF in response to stimulation, which causes active fibroblasts to develop into myofibroblasts, which express the PDGF receptor (PDGFR). The activation of the PDGF/PDGFR pathway drives the proliferation and migration of HSC and promotes ECM deposition, all of which advance the course of fibrosis (Klinkhammer et al., 2018). Liu et al. employed molecular docking and ion resonance biosensors to illustrate the strong binding affinity between PDGFR-β and *SalB*, and *SalB* relieves HF by inhibiting the PDGFR-β signaling pathway and inducing HSC apoptosis, and inflammatory reaction of HSC (Liu et al., 2023b). This suggests that Sal B exerts anti-fibrotic effects by directly targeting the PDGFRβ signaling cascade.

#### 2.1.3 Hedgehog

The hedgehog (Hh) pathway is thought to be connected with the level of fibrosis and has the capacity to activate HSC (Choi et al., 2009; Bar-Gal et al., 2012). Epithelial-mesenchymal transition (EMT) is a key process in which epithelial cells undergo phenotypic changes from an epithelial state to a mesenchymal state (Zhao et al., 2016). *SalB* effectively suppresses EMT in activated HSC by inducing microRNA (miR)-152 via the Hh signaling pathway, which in turn causes methyltransferase1 to be downregulated and Patched1 to be demethylated (Yu et al., 2015). Furthermore, the importance of EMT stimulated by Hh in the pathophysiology of HF has been shown in an increasing number of studies (Song et al., 2019; Zhang et al., 2022).

#### 2.1.4 Wnt

HF is dependent on the Wnt signaling system, which coordinates complex cell signaling networks, promotes HSC proliferative activation, and interacts cooperatively with other pro-fibrotic factors (Wang J. N. et al., 2018; Wang et al., 2024). Yu et al. discovered that *SalB* inhibited the Wnt/β-catenin signaling pathway, downregulated the production of miR-17-5p, and reduced the expression of α-SMA and ECM to limit the activation of HSC (Yu et al., 2016). Moreover, the specific mechanism of HF delay following *SalB* treatment is due to upregulated LincRNA-p21’s inhibition of HSC proliferation through the Wnt/β-catenin pathway (Yu et al., 2017). These findings identify the Wnt signaling pathway as potentially important for therapeutic targets in HF.

#### 2.1.5 NF-κB

The inflammatory response is mostly regulated by nuclear factor-κ-gene binding (NF-κB), a transcriptional regulator that is activated in chronic liver disorders and that promotes the production and secretion of pro-inflammatory cytokines (Karin and Ben-Neriah, 2000). Furthermore, the main ways that NF-κB contributes to HF are through controlling hepatocellular damage, altering inflammatory signaling pathways, and controlling fibrotic responses in HSC (Luedde and Schwabe, 2011). Wang et al.‘s study showed that *SalB* attenuated HF in mice by inhibiting the proliferation and activation of HSC by regulating the miR-6499-3p/LncRNA-ROR-mediated NF-κB signaling pathway (Wang et al., 2022). Additionally, *SalB* exhibited a dose-dependent reduction in the activation of nucleolus NF-κB, accompanied by an increase in cytoplasmic NF-κB levels (Wang et al., 2012). Thus, the regulation of the NF-κB pathway is one of the effective pathways of anti-HF (Basso et al., 2021; Ciceu et al., 2021).

#### 2.1.6 FGF

The fibroblast growth factor (FGF) family is involved in cellular proliferation and differentiation, angiogenesis, wound healing, and tissue regeneration (Degirolamo et al., 2016). FGF19 is an endocrine gastrointestinal hormone that regulates the metabolism of bile acids and possesses anti-fibrotic properties (Hirschfield et al., 2019). Several studies have reported that the FGF19/FGF Receptor4 (FGFR4) signaling pathway exerts an anti-fibrotic effect primarily through the inhibition of proliferative activation in HSC (Zhou et al., 2017; Gadaleta et al., 2018; Hirschfield et al., 2019). *SalB* exhibits the ability to upregulate the FGF19/FGFR4 pathway, which was disrupted by lipopolysaccharide (LPS) treatment, with the result that HSC activation and proliferation are inhibited (Tian et al., 2021). Given these functions, the endocrine FGF has therapeutic potential for inhibiting HSC.

#### 2.1.7 UGCG

The development of HF is closely associated with the overexpression of UDP-glucose ceramide glucosyltransferase (UGCG) in several chronic liver illnesses (Li et al., 2021). *SalB* considerably inhibits the progression of HF by inhibiting collagen deposition and HSC activation (Li et al., 2023). This is accomplished by the inhibition of UGCG by *SalB*, the additional reduction of immune cell infiltration brought on by carbon tetrachloride, the downregulation of α-SMA and CoI, and the suppression of phosphorylated histone, a hallmark of hepatic DNA damage.

**FIGURE 3:**
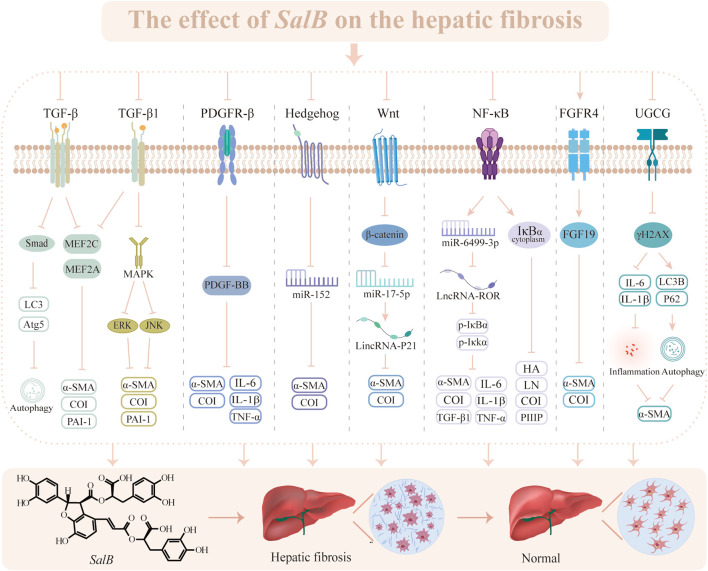
The effect of *SalB* on the hepatic fibrosis. Transforming growth factor-β (TGF-β), microtubule-associated protein light chain 3 (LC3), autophagy-related gene 5 (Atg5), myocyte enhancer factor 2 (MEF2), α-smooth muscle actin (α-SMA), collagen type I (CoI), plasminogen activator inhibitor-1 (PAI-1), mitogen-activated protein kinase (MAPK), extracellular regulated protein kinases (ERK), c-Jun N-terminal kinase (JNK), platelet-derived growth factor receptor-β (PDGFR-β), platelet-derived growth factor subunit B (PDGF-BB), interleukin-6 (IL-6), interleukin-1β (IL-1β), tumor necrosis factor α (TNF-α), microRNA (miR), wingless/Integrated (Wnt), nuclear factor-κ-gene binding (NF-κB), inhibitor of nuclear factor-κBα (IκBα), inhibiting kappa B kinase α (IκKα), hyaluronic acid (HA), laminin (LN), procollagen III peptide (PIIIP), fibroblast growth factor receptor 4 (FGFR4), fibroblast growth factor 19 (FGF19), UDP-glucose ceramide glucosyltransferase (UGCG), phosphorylation of Histone H2A Family Member X (γH2AX).

Based on the above studies, we can find that *SalB* exhibits anti-HF properties by impeding HSC proliferation and activation as well as suppressing collagen accumulation through diverse mechanisms, including TGF, Wnt, PDGF, Hh, etc. (Figure 3). Since HF is a result of multiple variables, even though the role of HSC in HF progression is obvious, is *SalB* useful in treating other pathogenic factors? Furthermore, all of the latest research confirms the efficacy of *SalB* on a single target in the HF process; more investigation is required to determine how several pathways interact.

### 2.2 Pulmonary fibrosis

Pulmonary fibrosis (PF) is an aberrant ECM deposition and excessive fibroblast accumulation that causes a chronic, progressive alveolar illness (Strieter and Mehrad, 2009). Lung epithelial cells are damaged under the influence of various stimuli, which trigger activation of the fibrotic pathway and subsequent collagen deposition. Consequently, excessive mesenchymal stromal cells and ECM replace normal lung tissue, leading to the structural destruction of the alveoli, dyspnea as a result of diminished lung compliance, and ultimately respiratory failure or death (Glass et al., 2020; Wu et al., 2021; Lu et al., 2022).

#### 2.2.1 TGF-β

TGF-β is the most potent pro-fibrotic mediator, inducing ECM, EMT, and pro-fibrotic mediators’ production by promoting the binding of Smad2/3 to Smad4, and driving myofibroblast differentiation to promote the PF process (Hu et al., 2018; Kramer and Clancy, 2018). Research has elucidated that patients with PF can exhibit elevated expression of TGF-β in their fibroblasts and airway epithelium (Xu et al., 2016). TGF-β, which plays a vital role in the development of PF, can stimulate airway smooth muscle hyperresponsiveness, goblet cell hyperplasia, and increased mucin secretion through Smad signaling (Ouyang et al., 2010; Ojiaku et al., 2018). Liu et al. interfered with the bleomycin (BLM)-induced PF model with *SalB*. The mechanism of *SalB* in relieving PF involves inhibiting the TGF-β pathway, including alleviating inflammatory injury, reducing disruption of alveolar architecture and collagen deposition, and inhibiting myofibroblast differentiation and EMT (Liu et al., 2016). SalB is a key subtype of TGF-β1 that serves an essential part in PF pathogenesis. It can attenuate CoI, α-SMA, and endogenous TGF-β1 production and inflammatory processes while inhibiting TGF-β1-induced proliferation, differentiation, and fibroblast-to-myofibroblast transformation in PF (Zhang et al., 2014; Jiang et al., 2020). Thus, targeting the TGF-β signaling pathway has emerged as an efficient therapeutic strategy for PF.

#### 2.2.2 PAR1

Protease-activated receptor 1 (PAR1) promotion of PF is attributed to various pathways that promote mitosis and angiogenesis, modulate pulmonary vascular permeability, and stimulate fibroblast migration (Kaneider et al., 2007; Tressel et al., 2011). By down-regulating PAR1, *SalB* inhibits coagulation factors, activates the fibrinolytic system, dissolves excessive fibrin deposition, preserves the structural integrity of pulmonary tissues, and suppresses fibrous tissue proliferation (Zhang et al., 2021). Therefore, PAR1 may be a promising target for the prevention and treatment of PF.

#### 2.2.3 Nrf2

Compared to other organs, the lung is exposed to higher oxygen tension and is the main organ in direct contact with inhaled oxidants. When fibrotic lesions appear in lung tissue, the body’s antioxidants may not be sufficient to counteract the significant amounts of oxidants that result from an imbalance between oxidation and anti-oxidation (Amara et al., 2010; Gao et al., 2024). In the meantime, the activated inflammatory factors trigger the production of substantial quantities of pro-fibrotic cytokines, which exacerbate oxidative stress in pulmonary tissue and intensify fibrosis (Obayashi et al., 2000; Kliment and Oury, 2010). *SalB* decreases malondialdehyde (MDA), myeloperoxidase, and reactive oxygen species (ROS) levels and increases superoxide dismutase activity, moreover, it inhibits inflammatory factors and activates the nuclear factor erythroid 2-related factor 2 (Nrf2) pathway to suppress myofibroblast transdifferentiating (Liu et al., 2018; Lu et al., 2022). Furthermore, *SalB* attenuated alveolar wall congestion, inflammatory cell infiltration, and emphysema in PF rat lung tissues while exerting notable anti-fibrotic effects. The results showed that Nrf2 was absent in PF areas, while the SalB treatment could increase the expression of Nrf2 in lung tissues.

#### 2.2.4 MAPK and NF-κB

Chronic inflammation is a feature of PF, which is closely associated with tissue damage resulting from the fibrotic response (Paola et al., 2011). Increased microvascular permeability brought on by ROS causes inflammatory cells to migrate and exacerbates the inflammatory process (Hecquet and Malik, 2009). MAPK and NF-κB are involved in chronic inflammation and oxidative stress-induced hyperpermeability of endothelial cells. BLM encourages the release of ROS, enhances endothelial cell permeability, and mediates apoptosis. Subsequent investigations have shown that *SalB* protects endothelial cells from oxidative stress injury by up-regulating the expression of the tight junction gene, reducing the permeability of injured endothelial cells, and counteracting the increase in ROS caused by BLM through the MAPK and NF-κB signaling pathways (Liu et al., 2018). *SalB* exhibits multiple features of anti-oxidant, inflammation inhibition, and immune modulation, which are indispensable for the treatment of PF (Ho and Hong, 2011).

**FIGURE 4:**
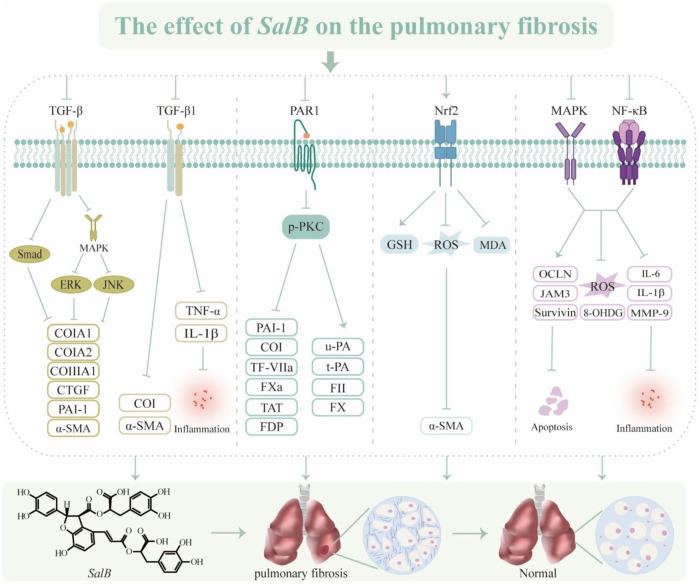
The effect of *SalB* on the pulmonary fibrosis. Transforming growth factor-β (TGF-β), mitogen-activated protein kinase (MAPK), extracellular regulated protein kinases (ERK), c-Jun N-terminal kinase (JNK), collagen type I (CoI), collagen type III (CoIII), connective tissue growth factor (CTGF), plasminogen activator inhibitor-1 (PAI-1), α-smooth muscle actin (α-SMA), tumor necrosis factor α (TNF-α), interleukin-1β (IL-1β), protease-activated receptor 1 (PAR1), phospho-protein kinase C (p-PKC), tissue factor (TF)/coagulation factor VII (TF-VIIa), activated coagulation factor X (FXa), thrombin-antithrombin complex (TAT), fibrinogen degradation product (FDP), urokinase type plasminogen activator (u-PA), tissue type plasminogen activator (t-PA), coagulation factor II (FII), coagulation factor X (FX), nuclear factor erythroid 2-related factor 2 (Nrf2), glutathione (GSH), reactive oxygen species (ROS), malondialdehyde (MDA), mitogen-activated protein kinase (MAPK), nuclear factor-κ-gene binding (NF-κB), occludin (OCLN), recombinant junctional adhesion molecule 3 (JAM3), 8-Hydroxy-2′-deoxyguanine (8-OHDG), interleukin-6 (IL-6), matrix metalloproteinase-9 (MMP-9).

In summary, one of *S. miltiorrhiza*’s most physiologically active ingredients, *SalB*, modulates TGF-β, PAR1, and oxidative stress to produce anti-fibrotic actions (Figure 4). But PF is a complex process that leads to the loss of structural lung tissue. EMT and circulating fibroblasts play a major role in controlling fibrosis, and their biological characteristics will be important to take into account when developing new treatment targets in the future to slow the progression of PF through *SalB*.

### 2.3 Cardiac fibrosis

Cardiac fibrosis (CF) is cardiac interstitial remodeling caused by excessive proliferative activation of fibroblasts and excessive deposition of secreted collagen matrix (Yang et al., 2024). Regardless of ejection fraction, CF is an essential event in the transition of cardiac function from compensatory to decompensated period and represents one of the significant factors contributing to mortality in patients with heart failure (Kanagala et al., 2019). The formation of this phenomenon is closely associated with the dysregulation of NF-κB, matrix metalloproteinase (MMP), fibronectin (FN), α-SMA, and connective tissue growth factor (CTGF), as well as the modulation of the renin-angiotensin-aldosterone system (RAAS) (Oatmen et al., 2020).

#### 2.3.1 NF-κB

NF-κB can participate in the occurrence and development of pathological cardiac remodeling by regulating the synthesis and release of ECM, cytokines, and chemokines (Wei et al., 2013; Zhang et al., 2016). Increasing evidence substantiates that NF-κB regulates the expression of vital genes in CF by intricate positive and negative feedback mechanisms, facilitating myofibroblast differentiation and promoting the fibrotic process (Li et al., 2020; Gong et al., 2021). Abnormal proliferation and differentiation of cardiac fibroblasts (CFB) can promote CF, and *SalB* inhibits Ang II-induced CFB activation and proliferation, as well as the fibrotic process by suppressing the expression of NF-κB and pro-fibrotic factors and ECM accumulation (Fan and Guan, 2016; Wang et al., 2018). More importantly, *SalB* also exhibits cardioprotective effects by protecting against LPS-induced cardiomyocyte injury through the Toll-like receptor 4 (TLR4)/NF-κB/Tumor Necrosis Factor α (TNF-α) pathway (Wang et al., 2011). In general, CF prevention and treatment techniques place a high priority on inhibiting the NF-κB signaling pathway via SalB’s effects (Wei et al., 2013).

#### 2.3.2 TRIM8

The tripartite motif-containing protein (TRIM) family has been acknowledged as an indispensable regulator in the process of myocardial injury (Yin et al., 2016). Studies have shown that heart tissue with strong TRIM32 expression further controls cystic fibrosis (Chen et al., 2016). Additionally, TRIM72 has been found to decrease fibroblast proliferation to promote fibrosis, while the knockdown of TRIM8 has been clarified to reduce myocardial ischemia-reperfusion injury (Zhao and Lei, 2016; Dang et al., 2020). By down-regulating TRIM8 expression, *SalB* effectively prevents oxygen radical generation and cardiomyocyte apoptosis via the TRIM8/recombinant glutathione peroxidase 1 (GPX1) signaling pathway both *in vivo* and *in vitro* (Lu et al., 2022). This elucidates a potential mechanism underlying *SalB*-mediated myocardial protection.

#### 2.3.3 MMP-9

The MMP-9 maintains the dynamic microenvironmental homeostasis of the ECM, involving collagen degradation associated with cardiovascular remodeling (Qin et al., 2010; Radosinska et al., 2017). In the context of myocardial infarction, MMP-9 prevents angiogenesis and exacerbates CF(Wang et al., 2021). During ventricular remodeling, MMP-9 activation upregulates fibrotic signaling and facilitates CF (Frangogiannis et al., 2002). *SalB* suppresses MMP-9 activity, diminishes the ratio of CoI/III, enhances cardiac contractility, and mitigates fibrosis (Jiang et al., 2010; Nandi et al., 2020). It is a competitive inhibitor of MMP-9 that guards against structural damage to the ventricles.

#### 2.3.4 AMPK

Adenosine monophosphate-regulated protein kinase (AMPK), a receptor of cellular energy status expressed in cardiomyocytes, is an essential part of CF (Harada et al., 2015). Research has illustrated that promoting the AMPK cascade can prevent cardiac hypertrophy, repair cardiac function, and impede the progression of fibrosis (Yun et al., 2014; Samanta et al., 2020). Ang II can stimulate collagen production and secretion, as well as fibroblast proliferation (Leask, 2015). *SalB* attenuates Ang II-induced ECM deposition via the forkhead box protein O1/miR-148a-3p axis and significantly reduces the levels of CoIA1, CoIII A1, α-SMA, CTGF, and ROS (Liu et al., 2023). By encouraging AMPK phosphorylation, SalB may counteract Ang II’s pro-fibrotic action on CF.

#### 2.3.5 IGFBP3

Insulin-like growth factor binding protein 3 (IGFBP3) has been evaluated to regulate cellular proliferation and migration and decrease activation of cardiac fibroblasts through epigenetic mechanisms (Ding et al., 2023). Besides, IGFBP3 miRNA and protein levels increase in CF. *SalB* inhibits the expression of IGFBP3, triggering the vascular endothelial growth factor receptor 2 (VEGFR2)/vascular endothelial growth factor A (VEGFA) pathway to enhance ventricular remodeling, angiogenesis, and collagen deposition linked to hyperglycemia, ultimately protecting cardiac function (Li et al., 2020).

**FIGURE 5:**
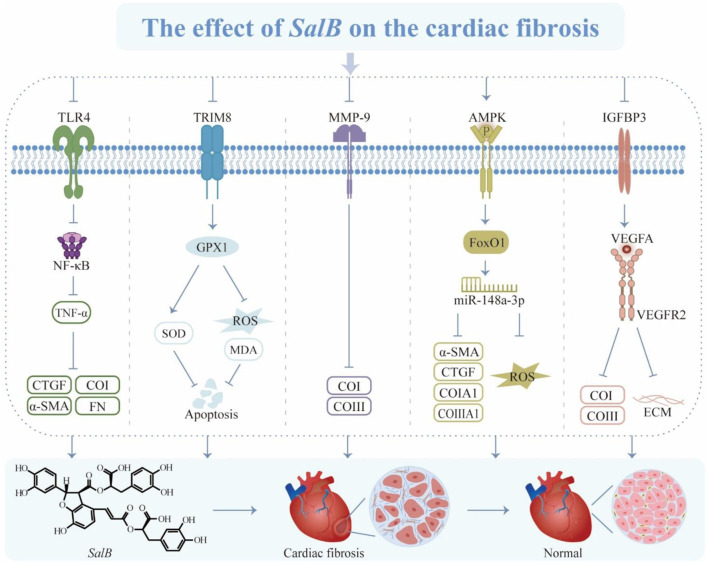
The effect of *SalB* on the cardiac fibrosis. Toll-like receptor 4 (TLR4), nuclear factor-κ-gene binding (NF-κB), tumor necrosis factor α (TNF-α), connective tissue growth factor (CTGF), α-smooth muscle actin (α-SMA), collagen type I (CoI), fibronectin (FN), tripartite motif 8 (TRIM8), glutathione peroxidase 1 (GPX1), superoxide dismutase (SOD), reactive oxygen species (ROS), malondialdehyde (MDA), matrix metalloproteinase-9 (MMP-9), collagen type III (CoIII), adenosine monophosphate-regulated protein kinase (AMPK), forkhead box protein 1 (FoxO1), connective tissue growth factor (CTGF), insulin-like growth factor binding protein 3 (IGFBP3), vascular endothelial growth factor A (VEGFA), vascular endothelial growth factor receptor 2 (VEGFR2), extracellular matrix (ECM).

Natural products have multitargeted actions that show promise in treating CF. *SalB* has the potential to serve as a complementary therapeutic agent for CF and exert an anti-fibrotic effect by modulating the formation of these substances (Figure 5). Even though their therapeutic effects have been demonstrated to be significant, the mechanisms of action are still unclear. Therefore, more research is required to understand the unique pathophysiology of CF and the synergistic process of *SalB*’s multi-point adjustments.

### 2.4 Renal fibrosis

Fibrous scar tissue generated by renal fibrosis (RF) replaces functional tissue, which impairs the regenerative ability of the kidneys and deteriorates renal function and tissue structure (Liu et al., 2018; Lin et al., 2023). RF is a prevalent feature observed in renal injury and the decline of renal function caused by various etiologies, serving as a common pathway for the progression of diverse chronic kidney diseases (Boor and Floege, 2012; Kramann et al., 2013). To avoid or delay the onset of chronic renal disease and improve the prognosis, it is imperative to target and block RF.

#### 2.4.1 TGF-β

TGF-β directly affects intrinsic renal cells, promoting cellular proliferation and triggering podocyte clearance and apoptosis of RTEC (López-Hernández and López-Novoa, 2012; Meng et al., 2015). Wang et al. found that *SalB* alleviated tubular fibrosis by inhibiting Smad2/3 phosphorylation as well as MMP-2 and MMP-9 activity via TGF-β1/Smad (Wang et al., 2010). *SalB* reverses TGF-β1-induced EMT, suppresses α-SMA expression, and facilitates the restoration of tubular epithelial structure to mitigate RF *in vitro* (Pan et al., 2011). A direct target of miRs is the TGF-β type II receptor, which regulates TGF-β signaling. SalB therapy increases miR-106b expression and inhibits TGF-β type II receptor expression to reduce TGF-β1-induced EMT (Tang et al., 2014). Despite the current dearth of efficacious medications for the treatment of RF, above numerous studies have manifested that targeting the TGF-β cascade represents an efficacious therapeutic approach for this condition.

#### 2.4.2 HPSE

Heparinase (HPSE) is an endonucleating β-D-glucuronidase that interacts with TGF-β to modulate the remodeling and degradation of the ECM, and the release of various cytokines (Ilan et al., 2006; Masola et al., 2014). The HPSE protein is expressed in RTEC and actively participates in the TGF-β-induced EMT in renal tubular cells (Masola et al., 2015). The integrity of the filtration barrier and the operation of glomerular filtration depends on this regulating mechanism (Masola et al., 2012; Rabelink et al., 2017). Tubular epithelial fibroblast transdifferentiation (TEMT) has been proposed as a viable therapeutic option to decrease RF since it stimulates the expression of several fibro-cytokines, which represent a critical mechanism in the development of RF (Li et al., 2004; Liu, 2010). *SalB* mitigates TEMT and has renal protective effects by down-regulating the expression of FGF2/TGF-β1/α-SMA via the HPSE/Syndecan-l axis (Hu et al., 2020).

#### 2.4.3 PDGF

PDGF is a profibrotic mediator that induces fibroblast activation and proliferation and promotes ECM contraction (Bonner, 2004). Numerous pro-fibrotic mediators, such as TGF-β, TNF-α, and FGF, exhibit PDGF-associated pro-fibrotic activity (Chaudhary et al., 2007). At locations where renal interstitial fibrosis occurs, there is an upregulation of PDGF-C expression, which stimulates PDGFRα to further the fibrotic process (Eitner et al., 2008; Folestad et al., 2018; Li et al., 2021). The efficacy of *SalB* in reducing renal inflammation and CTGF production, improving renal function, and inhibiting the progression of RF through the PDGF-C/PDGFRα pathway has been well-documented (Yao et al., 2022). In consequence, targeting PDGF with *SalB* presents a promising and effective new strategy for treating RF.

#### 2.4.4 PTEN/AKT

Phosphatase Tensin Homolog (PTEN) dephosphorylates protein kinase B (AKT), which inhibits pro-fibrotic signaling pathways and epithelial cell transdifferentiating; furthermore, RF is attenuated through the activation of AKT (Lan and Du, 2015; An et al., 2022). The administration of *SalB* has been shown to enhance renal function and prevent fibrosis by inhibiting Zeste gene enhancer homolog-2 (EZH2) and H3 lysine 27 trimethylation and down-regulating FN and α-SMA expression through the PTEN/AKT pathway (Lin et al., 2023).

#### 2.4.5 NLRP3

Nucleotide-binding oligomerization domain-like pyrin domain-containing protein 3 (NLRP3) is becoming increasingly implicated in the regulation of RF, as evidenced by the significant upregulation of its expression in renal fibrotic tissue (Ke et al., 2018; Wu et al., 2018; Mulay, 2019; Zhang and Wang, 2019). Moreover, NLRP3 influences RTEC apoptosis through mitochondrial connections in addition to promoting the advancement of the disease by inducing an inflammatory reaction (Qi and Yang, 2018). The effect of enhancing RTEC viability and mitigating cellular damage and apoptosis of *SalB* by modulating the TLR4/NF-κB/NLRP3 pathway to increase mitochondrial membrane potential levels (Zhang and Wang, 2019). Further, activation of the NLRP3 cascade promotes RF through RTEC pyroptosis (Hutton et al., 2016; Miao et al., 2019). To lessen renal injury, *SalB* enhances the nuclear accumulation of Nrf2 and suppresses NLRP3 activation, oxidative stress, and Caspase-1/Gasdermin D-mediated cellular pyroptosis via the Nrf2/NLRP3 pathway (Pang et al., 2020). According to the information above, *SalB* suppression of NLRP3 regulates RF and may be a useful target for treating RF.

#### 2.4.6 PI3K/AKT and Sirtuin1

The autophagy-lysosomal pathway is responsible for maintaining the metabolic balance of the ECM by sequestering FN within the cytosol and activating lysosomes for degradation in fibrosis (Guo et al., 2020). Autophagy stimulation can decrease synthesis, accelerate ECM degradation, and alleviate RF (Guo et al., 2020). SalB controls the important autophagy-mediating factor miR-145-5p to safeguard renal function. The mechanism is activating cellular autophagy, reducing cell proliferation, inflammation, and immune deposition via the phosphatidylinositol-3-kinase (PI3K)/AKT pathway (Chen et al., 2022a). *SalB* can also ameliorate autophagy and upregulate Sirtuin1, which can both lessen renal pathological damage and improve renal insufficiency (Pan et al., 2011; He et al., 2020). Additionally, it inhibits the expression of fibrogenic factors. Consequently, *SalB* as an autophagy mediator can protect renal function and ameliorate RF injury.

#### 2.4.7 Apoptosis

While the suppression of apoptosis ameliorates renal interstitial fibrotic lesions, the degree of apoptosis of RTEC corresponds with the grade of fibrosis, the index of renal interstitial damage, and the degree of renal function impairment (Docherty et al., 2006; Teteris et al., 2007). *SalB* therapy reduced ROS levels and lessened cellular damage brought on by endoplasmic reticulum stress (ERS) activation. It also suppressed the production of proteins linked to apoptosis, such as p-JNK, C/EBP homologous protein, and BCL2-associated X/B-cell lymphoma-2 (Dong et al., 2021). Intracellular free fatty acid-mediated lipotoxicity induces apoptosis in RTEC and contributes to fibrosis. This lipotoxicity is attenuated by *SalB*, which reduces apoptosis and tubular injury by inhibiting the activation of ERS and apoptotic markers (Mai et al., 2020).

**FIGURE 6:**
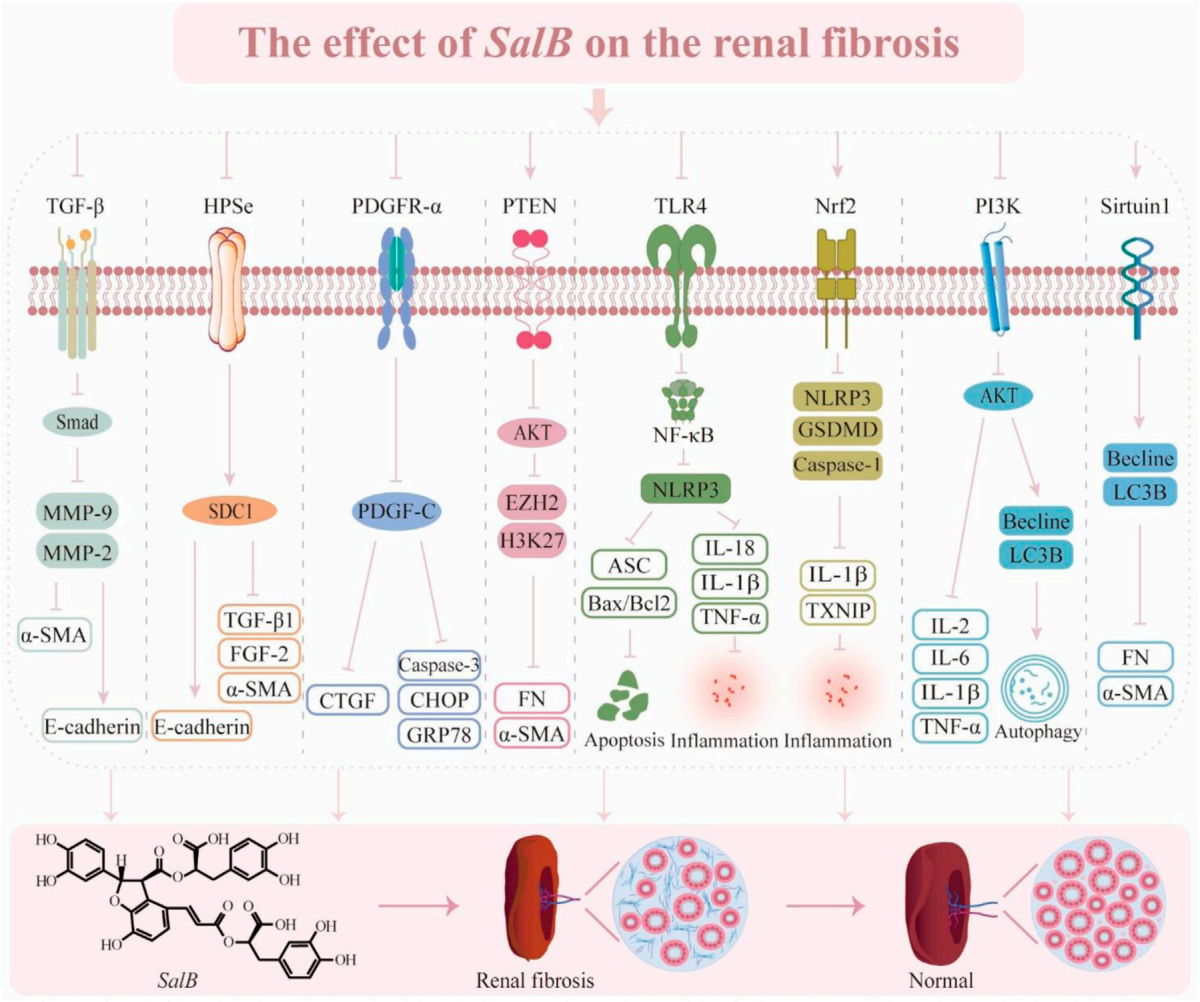
The effect of *SalB* on the renal fibrosis. Transforming growth factor-β (TGF-β), matrix metalloproteinase-9 (MMP-9), matrix metalloproteinase-2 (MMP-2), α-smooth muscle actin (α-SMA), heparinase (HPSe), syndecan-1(SDC1), fibroblast growth factor2 (FGF 2), platelet-derived growth factor receptor α (PDGFR-α), platelet-derived growth factor C (PDGF-C), connective tissue growth factor (CTGF), C/EBP homologous protein (CHOP), glucose regulated protein 78 (GRP78), phosphatase tensin homolog (PTEN), protein kinase B (AKT), enhancer of zeste homolog-2 (E2H2), histone H3 lysine 27 (H3K27), fibronectin (FN), toll-like receptor 4 (TLR4), nuclear factor-κ-gene binding (NF-κB), nucleotide-binding oligomerization domain-like pyrin domain containing protein 3 (NLRP3), apoptosis-associated speck-like protein containing a CARD (ASC), bcl-2 assaciated x protein (Bax), b-cell lymphoma-2 (Bcl2), interleukin-18 (IL-18), interleukin-1β (IL-1β), tumor necrosis factor α (TNF-α), nuclear factor erythroid 2-related factor 2 (Nrf2), gasdermin D (GSDMD), hioredoxin-interacting protein (TXNIP), phosphatidylinositol 3-kinase (PI3K), interleukin-2 (IL-2), interleukin-6 (IL-6), microtubule-associated protein light chain 3B (LC3B).

*SalB* affects the fibrosis of renal tubular epithelial cells (RTEC) by inhibiting various cytokines (PTEN, Ang II, TGF-β, EZH2, NF-κB, PDGF-C, CTGF) and pathways (cellular pyroptosis, autophagy, and apoptosis) (Figure 6). However, the pathophysiology of renal fibrosis is still poorly known, and renal fibrosis involves the dynamic process of various renal cells and inflammatory cells. Clinical research is lacking from the small number of cellular and animal tests that have been done so far, and several of the signaling pathways remain unclear.

### 2.5 Skin fibrosis

Hypertrophic scar is a prevalent yet inadequately resolved fibrotic dermatological condition, presenting as elevated, erythematous, indurated, and non-elastic plaques (Finnerty et al., 2016). They are among the most common side effects that arise when cutaneous damage heals. Targeting its downstream effector CD36, the SalB intervention lowers reticular fibroblast numbers and fibrogenic factors, which in turn inhibit cell proliferation and the production of skin scars (Griffin et al., 2021).

The pathogenesis of systemic sclerosis (SSc) is associated with endothelial damage, fibroproliferative vasculopathy, and fibroblast dysfunction (Taflinski et al., 2014; Garrett et al., 2017). TGF-β is a highly potent inducer of cell proliferation and collagen synthesis (Kim et al., 2015). *SalB* decreases skin thickness and collagen deposition by regulating the TGF-β/Smad and MAPK/ERK pathways, while down-regulating levels of CTGF, FN1, PAI-1, and α-SMA (Liu et al., 2019).

### 2.6 Epidural fibrosis

Epidural fibrosis (EF) is formed by excessive deposition of scar tissue in the epidural space following lumbar laminectomy (Zhang et al., 2013). Postoperative back and lower extremity pain is caused by the proliferation and adherence of scar tissue to the dura mater, as well as fibrosis around and enclosing the nerve roots (Fritsch et al., 1996; Ross et al., 1996). EF is therefore one of the main causes of the syndrome known as “failed lumbar spine surgery.” Inhibition of vascular regeneration and over-proliferation of fibroblasts is a promising strategy for reducing EF (Zhang et al., 2015). *SalB* inhibits the angiogenic factor VEGF, which mitigates fibroblast proliferation and blood vessel infiltration, reduces tissue adhesion, and prevents epidural scarring (Chen et al., 2016).

### 2.7 Frozen shoulder

Current treatments for arthrofibrosis are limited in terms of efficacy and diversity (Blessing et al., 2019). Fibro-proliferative tissue fibrosis is regarded as the primary pathological process underlying frozen shoulder (FS) (Challoumas et al., 2020). *SalB* has been found to have anti-inflammatory and anti-fibrotic effects in the treatment of FS. *SalB* inhibits the CD36-mediated PI3K/AKT pathway, downregulates the expression of fibrosis-associated molecules such as CoI, CoⅢ, FN, and α-SMA, and blocks the pathologic fibrotic process *in vitro* and *in vivo* (Yan et al., 2023).

### 2.8 Retinal fibrosis

Retinal fibrosis is a vascularized disease with excessive deposition of large numbers of immune cells, myofibroblasts, and ECM (Little et al., 2018; Tenbrock et al., 2022). A sustained low-grade inflammatory response drives the formation of fibrosis-associated lesions, facilitating the release of pro-fibrotic factors and the deposition of fibrotic tissue. In addition, pro-angiogenic astrocytes and microglia also contribute to the development of fibrotic lesions by producing VEGF and FN (Uemura et al., 2006). *SalB* attenuated the activation and proliferation of retinal microglia and astrocytes induced by amyloid β-protein deposition, along with the release of inflammatory factors through the β-Secretase one pathway (Wang et al., 2023). This increased dark-adapted and light-adapted amplitudes in mice restored the structure and function of the retina, and was associated with the reduction of ganglion cell apoptosis, attenuation of neuronal damage caused by oxidative stress, and restoration of mitochondrial function (Tian et al., 2008; He et al., 2018). According to other research, *SalB* protects retinal pigment epithelial cells from oxidative stress damage and H2O2-induced apoptosis while also restoring cell viability (Liu et al., 2016). These effects are mainly attributed to the activation of Nrf2.

### 2.9 Pancreatic fibrosis

Pancreatic fibrosis is a prominent morphological alteration observed in chronic pancreatitis, which is a multifaceted pathological process driven by the activation of pancreatic stellate cells (PSC) and distinguished by enhanced fibroblast proliferation and deposition of ECM (Singh et al., 2019; Beyer et al., 2020). Therefore, impeding the activation and proliferation of PSC is considered an approach for anti-pancreatic fibrosis therapy. *SalB* attenuates fibrotic damage, inhibits PSC activation and proliferation, and lowers the concentration of MDA in pancreatic tissues, all of which mitigate pathological harm (Lu et al., 2009).

### 2.10 Oral submucous fibrosis

Oral submucous fibrosis (OSF) is a chronic inflammatory disease identified by aberrant collagen deposition and progressive fibrosis of the subepithelial connective tissue in the oral submucosa (Utsunomiya et al., 2005). OSF is also a disorder of collagen metabolism caused by a disturbed balance of ECM synthesis and degradation. *SalB* inhibits the transcription of pro-collagen genes CoI and CoⅢ, and the expression of CTGF, IL-1β, and IL-6 by modulating the AKT, MAPK/ERK, and TGF-β/Smad signaling pathways (Dai et al., 2015a). It has notable anti-fibrotic action and decreases collagen deposition as well.

**FIGURE 7:**
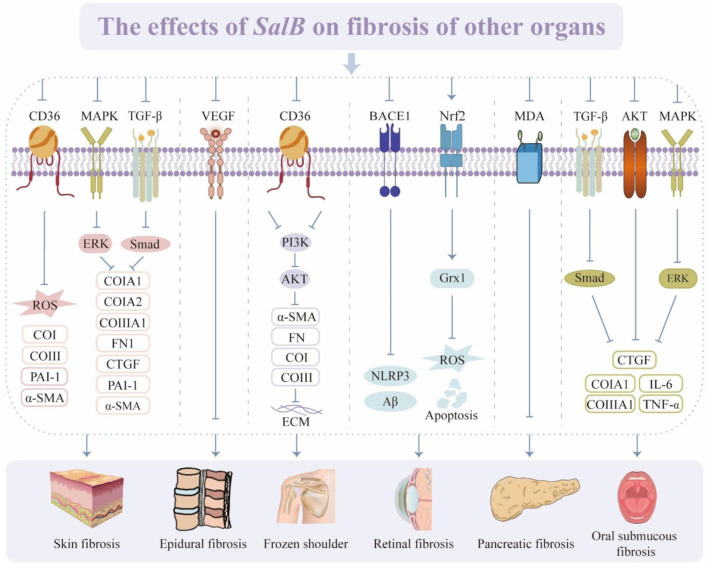
The effects of *SalB* on fibrosis of other organs. Recombinant Cluster of Differentiation 36 (CD36), reactive oxygen species (ROS), collagen type I (CoI), collagen type III (CoIII), plasminogen activator inhibitor-1 (PAI-1), α-smooth muscle actin (α-SMA), mitogen-activated protein kinase (MAPK), transforming growth factor-β (TGF-β), extracellular regulated protein kinases (ERK), fibronectin (FN), connective tissue growth factor (CTGF), vascular endothelial growth factor (VEGF), phosphatidylinositol 3-kinase (PI3K), protein kinase B (AKT), extracellular matrix (ECM), recombinant beta-site APP cleaving enzyme 1 (BACE1), nucleotide-binding oligomerization domain-like pyrin domain containing protein 3 (NLRP3), nuclear factor erythroid 2-related factor 2 (Nrf2), glutaredoxin 1 (Grx1), malondialdehyde (MDA), interleukin-6 (IL-6), tumor necrosis factor α (TNF-α).

The abovementioned studies validate the great potential of *SalB* in fibrosis of skin, pancreas, oral mucosa, and other tissues (Figure 7). However, further research is required to fully understand the role of *SalB* in these tissues to produce relevant and appropriate data for therapeutic trials and the interpretation of findings.

## 3 Safety and adverse effects

*SalB* has many biological benefits, such as anti-inflammatory, anti-oxidant, anti-fibrotic, and enhanced perfusion. It is extensively used in phenolic acids. Thus, the safety and adverse drug reactions of *SalB* have gradually become the focus of researchers’ attention. Compared to the equivalent doses of caffeic acid and ferulic acid, *SalB* does not induce the production of ROS or oxidative stress in the walls of small veins, preventing an imbalance in oxidative and anti-oxidant mechanisms (Du et al., 2017). Furthermore, a concentration of SalB of less than 300 mg/kg won't harm pregnant rats; at 100 mg/kg, SalB won't harm developing embryos or cause genotoxicity. SalB was tested for toxicity at a high dose of 750 mg/kg via the tail vein, and the findings point to SalB’s safety (He et al., 2023). According to another study, nebulized inhalation of SalB as a dry powder inhalation formulation demonstrated satisfactory mobility and no mortality in rat models. No significant side effects were noted in a randomized, double-blind clinical trial of SalB at single ascending doses and multiple ascending doses (Cheng et al., 2023). These findings suggest a favorable safety profile for *SalB*. In addition, the rate of fibrosis stage reversal was 36.67%, the rate of inflammation remission was 40.0%, and the serum fibrosis markers were significantly improved after 6 months of continuous use of SalB tablets (30 mg/tablet, 2 tablets once, three times a day) in the treatment of patients with hepatic fibrosis combined with consolidated hepatitis B. Furthermore, all members of the SalB group displayed normal test results and no adverse responses, indicating a high level of safety (Liu et al., 2002). The aforementioned research has established the safety of SalB in comparison to other synthetic medicines. To determine the optimal supplemental dosage and duration for treating fibrosis in different organs, as well as the possibility of hepatorenal toxicity when used over an extended period of time, more preclinical research is still required to support the data, and clinical studies are needed to produce high-caliber evidence-based research.

## 4 Drug interactions

Drug interaction (DI) between *SalB* and several other medications has been reported. For instance, the co-administration of tanshinone and polyphenol extract contributed to an increase in the area under the plasma concentration-time curve and a decrease in the total plasma clearance of *SalB* (Guo et al., 2008). When aspirin is taken together, *SalB* has a synergistic effect that inhibits CD62p, a molecular marker of platelet activation and extends *SalB*’s half-life by preventing the liver metabolizing enzyme catechol-O-methyltransferase (COMT) from being active (Cao et al., 2021). *SalB* also raises plasma levodopa levels after extended usage and prevents levodopa from being methylated via COMT. The activity of *SalB* was further enhanced in combination with ferulic acid (Chen et al., 2022). Nonetheless, the majority of the recent research on *SalB*’s DI focuses on synergistic potentiation rather than minimizing harmful side effects. Additionally, little is known about how *SalB* interacts with anti-fibrotic medications. Consequently, further studies are required to clarify these possible connections.

## 5 Different dosage forms of *SalB*

The absolute bioavailability of *SalB* is only 3.9% after oral administration of 50 mg/kg *SalB* to rats (Zhou et al., 2009). Another study found that dogs fed 180 mg/kg *SalB* orally had an AUC of 1680 ± 670 ng/mL h and a bioavailability of only 1.07% ± 0.43% (Gao et al., 2009). The main reasons for this include the enterohepatic circulation phenomena, hepatic first-pass effect, and inadequate permeability (Zhang et al., 2004a; 2004b; Wu et al., 2006; Zhou et al., 2009; Li et al., 2019). This limits *SalB*’s effectiveness and absorption in the gastrointestinal system and makes it less useful for clinical use. Researchers have manipulated *SalB* into several formulations to maximize its medicinal efficacy and increase its bioavailability. *SalB* was transformed into a lyophilized powder and dissolved in sodium chloride injection for intravenous infusion. Pharmacokinetic analysis revealed that there was no discernible accumulation of *SalB* in the body as the maximum plasma concentration and area under the curve (AUC) rose proportionately with increasing doses (Bi et al., 2016). Pulmonary administration via dry powder inhaler circumvents first-pass effects and diminishes enzymatic degradation reactions (Newman, 2017). The drug concentration in the lungs was elevated by the dry powder inhalation formulation of *SalB*, which had an AUC (0–1) that was almost 2099.12 times higher than that of intravenous treatment (Jiang et al., 2021). Loading *SalB* into a solid self-micro emulsifying drug delivery system for lipid delivery prepared by coagulating the liquid excipient into a powder (Bi et al., 2016). Higher bioavailability is made possible by this technique, which also guarantees a more effective medication delivery mechanism. Nanotechnology has made a major impact on the advancement of medicine, as nano-encapsulated natural products have better safety, stability, and efficacy. Compared to previous formulations, *SalB*-loaded PEGylated liposomes at an active dose of 100 mg/kg showed higher than 4-fold plasma concentrations (Isacchi et al., 2011). *SalB*’s bioavailability can be significantly increased by using different dosage forms. However, less research has been done on fibrosis treatment. Anti-fibrotic drug development at the nexus of biology, pharmacy, and materials science may take an important turn in this direction in the future.

## 6 Discussion

Natural products are crucial sources of modern drug discovery and play a pivotal role in disease prevention and treatment. With the advancements in molecular biology and modern chromatographic separation techniques and the mechanisms of the fibrotic process constantly being elucidated, an increasing number of natural anti-fibrotic products are being discovered. *SalB* is considered a promising treatment in the field of combating fibrotic diseases. The details of its anti-fibrotic effects on multiple tissues and organs through activation of different signaling pathways and modulation of multiple targets are shown in Table 1. While significant progress has been achieved in current research on the pathogenesis of fibrosis, there are several challenges to translating this research into clinical practice.

**TABLE 1:** Anti-fibrotic effects of *SalB*.

Disease	Animal/cell model	Dosage and duration	Mode of administration	Described effects	Pathways	Refer
Hepatic fibrosis	*In vivo*: DEN induced Wistar rat	15, 30 mg/kg; 12weeks	Gavage	α-SMA↓, CoI↓, ERK↓, JNK↓, p38↓,Smad2C↓,Smad2L↓, Smad3C↓, Smad3L↓,PAI-1↓	TGF-β/Smad; MAPK	Wu et al. (2019)
	*In vitro*: TGF-β1 stimulated HSC	25, 50, 100μM; 24 h	—			
	*In vivo*: DMN induced SD rat	12.5 mg/kg; 3weeks	Gavage	α-SMA↓, MEF2A↓, MEF2C↓, MARK↓	TGF-β/MEF2	Zhang et al. (2019)
	*In vitro*: TGF-β1 stimulated HSC	1.10 μM; 24 h	—			
	*In vitro*: TGF-β1 stimulated JS1 and LX2	10 μM; 24 h	—	LC3BII↓,CoI↓,α-SMA↓,Atg5↓,ERK↓, JNK↓	TGF-β1/MAPK	Jiang et al. (2022)
	*In vivo*: CCL_4_ induced C57BL/6J mice	25,50 mg/kg; 4weeks	Gavage	CoI↓, α-SMA↓, PDGFR β↓, IL-1β↓, IL-6↓, TNF-α↓, TGF-β↓,COX-2↓	PDGFRβ	Liu et al. (2023a)
	*In vitro*: T6 and LX-2 cell lines	5,10,20,40.80 μM; 24 h,48 h	—			
	*In vivo*: CCL_4_ induced C57BL/6J mice	100 mg/kg; 8weeks	Gavage	miR-152↓, CoI↓, α-SMA↓, E-cadherin↑, PTCH1↑, DNMT1↓	Hh	Yu et al. (2015)
	*In vitro*: HSC	10 μM; 48 h	—			
	*In vitro*: HSC	10 μM; 48 h	—	miR-17-5p↓, LincRNA-p21↑, CoI↓, α-SMA↓	Wnt/β-catenin	Yu et al. (2017)
	*In vivo*: CCL_4_ induced BALB/c mice	50 mg/kg; 4weeks	Gavage	NF-κB↓, miR-6499-3p↑, LncRNA-ROR↓, p-IκKα↓,p-IκBα↓, CoI↓, TGF-β1↓,α-SMA↓, IL-6↓, IL-1β↓, TNF-α↓	NF-κB	Wang et al. (2022)
	*In vitro*: T6 and LX-2 cell lines	40,80 μM; 24 h,48 h,72 h	—			
	*In vivo*: CCL_4_ induced SD rats	10.20 mg/kg; 6weeks	Intraperitoneal injection	NF-κB (nucleolus)↓, NF-κB (cytoplasm) ↑, IκBα(cytoplasm)↑,HA↓,LN↓, CoI↓,PⅢP↓	NF-κB/IκBα	Wang et al. (2012)
	*In vitro*: LPS stimulated LX2	1–5 μM; 72 h	—	FGFR4↑, FGF19↑, CoI↓, α-SMA↓	FGFs	Tian et al. (2021)
	*In vivo*: CCL_4_ induced C57BL/6J mice	30 mg/kg; 4weeks	Intraperitoneal injection	γH2AX↓, IL-1β↓, IL-6↓, α-SMA↓, LC3B↑, P62↑	UGCG	Li et al. (2023)
	*In vitro*: LX2, WRL68	25, 50μM; —	—			
Pulmonary fibrosis	*In vivo*: BLM induced C57BL/6 mice	10 mg/kg; 3weeks	Intraperitoneal injection	ERK↓, JNK↓, CoIA1↓, CoIA2↓, CoⅠⅠⅠA1↓, CTGF↓,α-SMA↓, PAI-1↓	TGF-β/Smad, MAPK	Liu et al. (2016a)
	*In vitro*: TGF-β stimulated MRC-5, A549, NIH/3T3	50 μg; 24 h	—			
	*In vitro*: TGF-β1 stimulated MRC-5	1,10uM; 24 h		TGF-β1↓, CoI↓,α-SMA↓	TGF-β1	Zhang et al. (2014)
	*In vitro*: TGF-β1 stimulated MRC-5	0,20,50,100,200,300,400,500,600,700,800,900 μg/mL; 24 h	—	COI↓,α-SMA↓,TNF-α↓, IL-1β↓	TGF-β1	Jiang et al. (2020)
	*In vitro*: LPS stimulated THP-1	0,10,25,50,75,100,125, 150,200 μg/mL; 48 h	—			
	*In vivo*: BLM induced SD rats	0.15,0.3,0.45 mg/kg; 28 days	Inhalation	PAR1↓, p-PKC/PKC↓,PAI-1↓, CoI↓, TF-VIIa↓,FXa↓, TAT↓, FDP↓, t-PA↑,u-PA↑, FII↑, FX↑	PAR1/p-PKC	Zhang et al. (2021)
	*In vivo*: BLM induced Wistar rats	20 mg/kg; 2weeks	Intravenous injection	Nrf2↑,ROS↓,GSH↑,MDA↓,α-SMA↓	Nrf2	Liu et al. (2018b)
	*In vitro*: TGF-β1 stimulated MRC-5	40μM; 48 h	—			
	*In vivo*: BLM induced SD rats	10 mg/kg; 4weeks	Inhalation	COIA1↓, COⅠⅠⅠA1↓, FN↓,iNOS↓, MDA↓,MPO↓,TGF-β1↓,IL-1β↓, IL-6↓, IL-18↓	—	Lu et al. (2022b)
	*In vitro*: TGF-β1 stimulated NIH-3T3	25,50,125 μg/mL; 48 h	—			
	*In vivo*: BLM induced C57BL/6 mice	10 mg/kg; 1weeks	Intraperitoneal injection	TUNEL↓, OCLN↑, Survivin↑, JAM3↑, ROS↓, 8-OHdG↓, IL-1β↓, IL-6↓, MMP-9↓	MAPK, NF-κB	Liu et al. (2018c)
	*In vitro*: H2O2 stimulated EAhy926	50 μg/mL; 12 h	—			
Cardiac fibrosis	*In vitro*: Ang II stimulated CFBs	12.5,25,50 μM; 24 h	—	NF-κB↓, CoⅠ↓, FN↓, α-SMA↓, CTGF↓	NF-κB	Wang et al. (2018a)
	*In vitro*: LPS stimulated cardiomyocytes	0.1,1,10 μM; 6 h	—	TLR4↓, NF-κB↓, TNF-α↓, LDH↓	TLR4/NF-κB/TNF-α	Wang et al. (2011)
	*In vivo*: I/R induced SD rats	20,40,60 mg/kg; 1weeks	Intraperitoneal injection	TRIM8↓, GPX1↑, SOD↑, ROS↓, MDA↓	TRIM8/GPX1	Lu et al. (2022a)
	*In vitro*: I/R stimulated AC16 cells	10, 25,50μM; 24 h	—			
	*In vivo*: ligation of the left anterior descending coronary artery induced SD rats	10 mg/kg; 2weeks	Intravenous injection	MMP-9↓, CoⅠ/ⅠⅠⅠ↓	MMP-9	Jiang et al. (2010)
	*In vivo*: Ang II induced BALB/c mice	200 mg/kg; 4weeks	Intravenous injection	p-AMPK↑, FoxO1↑, miR-148a-3p↑,α-SMA↓, CTGF↓, CoIA1↓, CoⅠⅠⅠA1↓, ROS↓	AMPK/FoxO1	Liu et al. (2023c)
	*In vitro*: Ang II stimulated FBs	12.5,25,50 μM; 2 h	—			
	*In vivo*: STZ induced C57BL/6J	15,30 mg/kg; 16weeks	Intraperitoneal injection	IGFBP3↓, VEGFA↑,VEGFR2↑, CoⅠ↓,CoⅠⅠⅠ↓, ECM↓	IGFBP3	Li et al. (2020a)
	*In vitro*: hypoxia incubator stimulated HUVECs	—	—			
Renal fibrosis	*In vivo*: HgCl_2_ induced SD rats	10 mg/kg; 9weeks	Gavage	TGF-β1↓, p-Smad2↓,p-Smad3↓, MMP-2↓,MMP-9↓,α-SMA↓, E-cadherin↑	TGF-β/Smads	Wang et al. (2010)
	*In vitro*: TGF-β1 stimulated HK-2	1.10μM; 24 h	—			
	*In vitro*: TGF-β1 stimulated HK-2	0.1,1,10,100 μM; 72 h	—	α-SMA↓, E-cadherin↑	TGF-β	Pan et al. (2011)
	*In vitro*: TGF-β1 stimulated HK-2	1.50 μM; 48 h	—	TGF-β↓,miR106b-25↑,α-SMA↓, E-cadherin↑	TGF-β	Tang et al. (2014)
	*In vivo*: unilateral ureteral obstruction (UUO) induced C57BL/6J	6.25,12.5.25 mg/kg; 2weeks	Intraperitoneal injection	HPSe↓, SDC1↑, α-SMA↓, TGF-β1↓, FGF-2↓, E-cadherin↑	HPSe/SDC1	Hu et al. (2020)
	*In vitro*: Ang II stimulated HK-2	0.1,1, 10 μM; 24 h	—			
	*In vivo*: UUO induced SD rats	12.5 mg/kg; 2weeks	Gavage	PDGFR-α↓, PDGF-C↓, CTGF↓, Caspase-3↓, CHOP↓,GRP78↓	PDGF-C/PDGFR-α	Yao et al. (2022)
	*In vitro*: HAS stimulated HK-2	20 μM; 48 h	—			
	*In vivo*: UUO/AAN induced C57/6J	10 mg/kg; 2/4weeks	Intraperitoneal injection	PTEN↑, AKT↓, EZH2↓,H3K27↓, FN↓,α-SMA↓	PTEN/AKT	Lin et al. (2023)
	*In vitro*: TGF-β1 stimulated NRK-49F	3,10,30 μM; 24 h	—			
	*In vitro*: Iopromide stimulated HK-2	10,50, 100 μM; 3 h	—	TLR4↓, NF-κB↓, NLRP3↓, ASC↓, Bax/Bcl2↓, IL-18↓, IL-1β↓, TNF-α↓	TLR4/NF-κB/NLRP3	Pei et al. (2022)
	*In vivo*: I/R induced Balb/c mice	50,100,200 mg/kg; 6weeks	Gavage	Nrf2↑, NLRP3↓, GSDMD↓, Caspase-1↓, IL-1β↓, TXNIP↓	Nrf2/NLRP3	Pang et al. (2020)
	*In vitro*: HK-2	1,5,10,20,40,80 μM; 24 h	—			
	*In vivo*: cBSA induced SD rats	100 mg/kg; 2weeks	Gavage	PI3K↓, AKT↓, Beclin1↑,LC3B↑, IL-2↓,IL-6↓, IL-1β↓, TNF-α↓	PI3K/AKT	Chen et al. (2022b)
	*In vitro*: LPS stimulated HMCs	—;24 h	—			
	*In vivo*: Adriamycin and unilateral nephrectomy induced SD rats	50,100,200 mg/kg; 6weeks	Gavage	Sirtuin1↑, Beclin1↑,LC3B↑, FN↓,α-SMA↓	Sirtuin1	He et al. (2020)
	*In vitro*: TGF-β1 stimulated HK-2	100 μM; 24 h	—			
	*In vitro*: Iopromide stimulated HK-2	10,50,100 μM; 3 h	—	ROS↓, p-JNK↓,CHOP↓,Bax/Bcl-2↓, caspase-3↓, GRP78↓	—	Dong et al. (2021)
	*In vivo*: HFD induced C57BL/6J	3,6.25,12.5 mg/kg; 4weeks	Intraperitoneal injection	Bax↓, Caspase-3↓,ATF4↓,CHOP↓, ATF6↓,IRE1α↓	—	Mai et al. (2020)
	*In vitro*: PA, TM, TG stimulated HK-2	1,10,100 μM; 24 h	—			
Skin fibrosis	*In vivo*: Tetracycline induced JUN mice	100 μg/mL	Intraperitoneal injection	CD36↓, ROS↓, CoI↓,COⅠⅠⅠ↓, PAI-1↓, α-SMA↓	CD36	Griffin et al. (2021)
	*In vitro*: human HTS fibroblasts	—	—			
	*In vivo*: BLM induced C57BL/6 mice	10 mg/kg; 3weeks	Intraperitoneal injection	Smad↓, ERK↓, CoI A1↓, CoI A2↓, CoⅠⅠⅠA1↓, FN1↓, CTGF↓,PAI-1↓,α-SMA↓	TGF-β/Smad MAPK/ERK	Liu et al. (2019)
	*In vitro*: TGF-β stimulated Human skin fibroblasts	50,100,150,200,250 μg/mL	—			
Epidural fibrosis	*In vivo*: laminectomy induced Wistar rats	10,30,50 mg/kg; 8 weeks	Gavage	VEGF↓	—	Chen et al. (2016a)
frozen shoulder	*In vivo*: molding plaster for 3 weeks induced SD rats	50 mg/kg; 3weeks	Intraperitoneal injection	CD36↓, PI3K↓, AKT↓,α-SMA↓, FN↓, CoI↓, COⅠⅠⅠ↓	CD36	Yan et al. (2023b)
	*In vitro*: synovial fibroblasts	0,20,50,100,200,300 μg/mL; 72 h	—			
Retinal fibrosis	*In vivo*: 5×FAD mouse	20 mg/kg; 12weeks	Gavage	BACE1↓, Aβ↓, NLRP3↓	BACE1	Wang et al. (2023)
	*In vitro*: H2O2 stimulated retinal pigment epithelium	50 μM; 24 h	—	Nrf2↑, Grx1↑, ROS↓	Nrf2	Liu et al. (2016b)
Chronic Pancreatitis	*In vivo*: TNBS induced SD rats	10 mg/kg; 8weeks	Gavage	MDA↓	—	Lu et al. (2009)
Oral Submucous fibrosis	*In vitro*: ANE stimulated MOMFs	0,3.125,6.25,12.5,25,25 50 μM; 24 h	—	TGF-β↓, Smad↓, AKT↓, ERK↓, CTGF↓, CoIA1↓, CoⅠⅠⅠA1↓, IL-6↓,TNF-α↓	TGF-β/Smad, AKT, ERK/MAPK	Dai et al. (2015b)

Adequacy of resources is a challenge for drugs derived from natural products. The problem of natural product resources can be greatly solved by synthesizing bioactive compounds having pharmacological properties using either biotic or abiotic approaches. Nevertheless, *SalB* extraction from the natural product *S. miltiorrhiza* is the main method to obtain *SalB*. However, according to some research, *SalB* can be produced artificially using biotechnological means: *rosmarinic acid*, the precursor compound for *SalB*, can be made into salvianolic acid by creating a full biometabolic pathway for it in a strain of *Saccharomyces cerevisiae*. This can be achieved by using endogenous enzymes in the yeast chassis cells in conjunction with an exogenous biosynthetic pathway (Di et al., 2013; Mateljak et al., 2017). While it is possible to produce *SalB* through a technological method, more research is necessary to determine whether the molecules produced through this process have the same pharmacological effects as *SalB* derived from natural sources.

One more problem that must be resolved before *SalB* can be used in the clinic is bioavailability. Due to SalB’s restricted oral bioavailability, attempts are now being made in research to find substitute formulations. Research is now being done on *SalB*’s lyophilized powder, dry powder inhaler, and lipid carrier alternatives. However, little is known about its use in fibrotic disorders. The effectiveness of *SalB* in treating fibrotic diseases has been confirmed. To enhance efficacy and improve bioavailability, future research endeavors ought to concentrate on the preparation of *SalB* into diverse dosage forms that are customized to the unique features of the disease.

The comprehension of fibrotic molecules is improved through modeling (Ricard-Blum and Miele, 2020). It is essential to create predictive animal models, primary human tissue culture systems, and virtualized medications based on particular genetic profiles of fibrotic patients due to the developments of modern technology, such as single-cell multi-omics. Before a medicine enters clinical trials, its effect on particular molecular disease phenotypes can be predicted.

One significant barrier to the development of anti-fibrotic medications is the lack of suitable biomarkers for identifying disease-specific traits (Wynn and Ramalingam, 2012). Therefore, methods for quickly, non-invasively, and precisely determining the anti-fibrotic efficacy of *SalB* are essential for its clinical application. Normal repair processes require the production of FN, the release of inflammatory agents, and the deposition of ECM during fibrosis. To avoid off-target toxicity, more research is needed to ascertain whether *SalB* can target key fibrotic components upstream and downstream and whether its use affects the normal repair process in any way. In addition, the majority of recent research on *SalB*’s anti-fibrotic action focuses mostly on one target or pathway. On the other hand, little is known about the ways in which various targets and pathways interact. Thus, in order to more thoroughly understand the overall regulation of *SalB*, we can conduct studies on the regulatory mechanism of the intricate network of interacting targets using techniques including single-cell sequencing, proteomics, transcriptomics, and metabolomics.

Few comprehensive clinical investigations have been conducted on the safety and side effects of *SalB*, despite multiple preclinical studies showing the drug’s anti-fibrotic efficacy in a variety of organs (Ma et al., 2017; Cheng et al., 2023). There is an urgent need to address these issues through large-scale clinical studies. Furthermore, selecting subjects for clinical trials is difficult due to the diversity of clinical conditions and the slow course of fibrosis. The development of successful anti-fibrotic therapeutics depends on defining reliable and valid predictors of the course of fibrotic disease and planning clinical studies with distinct clinical outcomes to typify patients prior to trial enrollment (Henderson et al., 2020).

## 7 Conclusion

*SalB* has garnered interest due to its diverse array of anti-fibrotic properties. Several studies have been done on *SalB*’s impact on fibrosis in both cells and animals. *SalB* may be able to mitigate the fibrotic process by modifying multiple aspects of immune cells, inflammatory factors, oxidative stress, ERS, and pyroptosis to lessen the release of fibrogenic factors, ECM over deposition, and fibroblast proliferation. It is reasonable to conclude that *SalB* may be a pleiotropic molecule exerting anti-fibrotic effects against numerous signaling cascades after carefully reviewing the studies on *SalB*’s effects on fibrosis. There are some limitations since the current study is a review that focuses on *SalB*’s involvement in fibrosis in various organs. Firstly, as the study is a review of the research that has been done, it falls short in terms of offering clinical evidence supporting *SalB*’s use in the treatment of fibrosis. Second, this review aims to shed light on the scope and efficacy of *SalB* in treating fibrotic diseases by describing and summarizing *SalB*’s effects on fibrosis in various organ tissues. It is necessary to reevaluate from a different angle whether *SalB*’s mechanism of action is similar for different fibrotic disorders or whether similar therapeutic targets are present. Lastly, the evidence that can be offered for this study is limited because there are not enough studies on *SalB* for the therapy of fibrosis in other organs, such as the skin, pancreas, retina, etc. All of these issues require further in-depth research, discussion, and augmentation in the future.

In conclusion, additional excellent, advanced basic and clinical research is required to fully comprehend how *SalB* affects fibrosis, to enhance *SalB*’s therapeutic benefits, particularly with regard to organ damage carried on by fibrosis, and to fulfill the intended purpose of targeted therapy.
